# Different injury patterns exist among patients undergoing operative treatment of isolated PCL, combined PCL/ACL, and isolated ACL injuries: a study from the Swedish National Knee Ligament Registry

**DOI:** 10.1007/s00167-022-06948-x

**Published:** 2022-03-31

**Authors:** Bálint Zsidai, Alexandra Horvath, Philipp W. Winkler, Eric Narup, Janina Kaarre, Eleonor Svantesson, Volker Musahl, Eric Hamrin Senorski, Kristian Samuelsson

**Affiliations:** 1 Department of Orthopaedics, Institute of Clinical Sciences, Sahlgrenska Academy, University of Gothenburg, Gothenburg, Sweden; 2 Department of Internal Medicine and Clinical Nutrition, Institute of Medicine, Sahlgrenska Academy, University of Gothenburg, Gothenburg, Sweden; 3 Department for Orthopaedic Sports Medicine, Klinikum rechts der Isar, Technical University of Munich, Munich, Germany; 4 Department of Orthopaedic Surgery, UPMC Freddie Fu Sports Medicine Center, University of Pittsburgh, Pittsburgh, USA; 5 Department of Health and Rehabilitation, Institute of Neuroscience and Physiology, Sahlgrenska Academy, University of Gothenburg, Gothenburg, Sweden; 6 Department of Orthopaedics, Sahlgrenska University Hospital, Mölndal, Sweden

**Keywords:** Posterior cruciate ligament, Knee ligament, Epidemiology, Injury mechanism, Ligament registry, Multiligament knee injury

## Abstract

**Purpose:**

To compare demographic characteristics and concomitant injury patterns in patients undergoing primary isolated posterior cruciate ligament reconstruction (PCL-R) and combined posterior cruciate ligament (PCL) and anterior cruciate ligament (ACL) reconstruction (PCL-R/ACL-R) with isolated ACL reconstruction (ACL-R) as a reference using data from the Swedish National Knee Ligament Registry (SNKLR).

**Methods:**

This cohort study based on the SNKLR comprised patients undergoing either PCL-R, ACL-R, or combined PCL-R/ACL-R between January 1, 2005 and December 31, 2019 in Sweden. Demographic and surgery-related data with regards to injury mechanism, concomitant intraarticular lesions and their treatment, neurovascular damage, and concomitant ligamentous injuries were extracted. Exclusion criteria included concomitant fractures of the femur, fibula, patella or tibia, and quadriceps or patellar tendon injury.

**Results:**

A total of 45,564 patients were included in this study. Isolated PCL-R, combined PCL-R/ACL-R, and isolated ACL-R were performed in 192 (0.4%), 203 (0.5%) and 45,169 (99.1%) patients, respectively. Sports were identified as the cause of 64% of PCL-Rs, 54% of PCL-R/ACL-Rs, and 89% of ACL-Rs, while a traffic-related mechanism was identified in 20% of PCL-Rs, 27% of PCL-R/ACL-Rs and 2% of ACL-Rs. Meniscus injury prevalence was 45% in ACL-Rs, 31% in PCL-R/ACL-Rs and 16% in isolated PCL-Rs (*p* < 0.001). Cartilage injuries were more common in PCL-R (37%) and PCL-R/ACL-R patients (40%) compared to ACL-R patients (26%, *p* < 0.001). Concomitant knee ligament injury was identified in 28–44% of PCL-R/ACL-R patients. Neurovascular injuries were present in 9% of PCL-R/ACL-Rs, 1% of PCL-Rs, and 0.3% of ACL-Rs (*p* < 0.001).

**Conclusion:**

Differences in injury mechanisms among patient groups confirm that operatively treated PCL tears are frequently caused by both traffic and sports. Cartilage and ligament injuries were more frequent in patients with PCL-R compared to ACL-R. Consequently, combined PCL and ACL tears should raise suspicion for concomitant knee lesions with clinical relevance during the operative treatment of these complex injuries.

**Level of evidence:**

III.

**Supplementary Information:**

The online version contains supplementary material available at 10.1007/s00167-022-06948-x.

## Introduction

Despite being considered a rare injury overall with a 1–6% estimated incidence, posterior cruciate ligament (PCL) tears are devastating knee injuries with a detrimental effect on subsequent knee function [7, 20, 26]. During the 2010s, several studies conducted on national knee ligament registries have provided novel information on injury patterns and clinical outcomes of operatively treated PCL injuries [3, 14, 16, 27]. A larger cross-sectional area and tensile strength of the PCL compared to that of the anterior cruciate ligament (ACL) renders it more resistant to tears, leading to PCL tears most commonly occurring during high-intensity sport and traffic-related traumas. Contrary to the traditional view that PCL tears are mainly the result of traffic accidents, the majority of operatively treated PCL injuries are caused by sports activity [14, 16, 22].

A non-operative approach is generally preferred for the initial treatment of PCL tears; however, isolated PCL tears are responsible for 38% of operatively treated PCL injuries in Scandinavia [16]. Although isolated PCL reconstruction (PCL-R) has demonstrated similar improvement in subjective outcomes as isolated ACL reconstruction (ACL-R), inferior preoperative knee function has been reported in patients who undergo PCL-R [3]. While these findings denote the detrimental effect of PCL tears on the knee joint, the exact factors responsible for inferior knee function and return to sport rate in this patient population are currently unknown [6]. Intra-articular pathology and concomitant knee ligament injuries are frequent findings [10, 16, 19, 24] with a well-established negative impact on knee function. However, a recent study from the Danish Knee Reconstruction Registry reported isolated PCL tears to display lower rates of damage to the menisci (19% vs. 46%) and articular cartilage (15% vs. 61%) compared to isolated ACL tears [14]. A more detailed understanding of the intraarticular injury patterns observed in subsets of PCL-R patients may provide an explanation for the inferior knee function observed in this population.

Consequently, a comprehensive investigation of the etiology, demographic characteristics, and concomitant injuries is necessary to expand the understanding of PCL tears contributing to the reported inferior functional outcomes. There is currently limited information with regards to the distribution of the specific knee ligaments injured in conjunction with the PCL in operatively treated combined PCL tears [13, 14, 16, 21]. Previous data from Scandinavia identified the ACL to be the most frequently injured knee ligament concurrent to PCL tears, but a more exhaustive investigation of ligament injury pattern is required to understand the potential impact of concomitant ligament injuries on functional outcomes following PCL-R [16].

Knowledge of frequently occurring knee injury patterns associated with PCL-R may help improve the surgical management of complex knee ligament tears. The purpose of this study was to explore and compare the demographic profile and concomitant injury patterns in patients undergoing primary isolated PCL-R, primary isolated ACL-R and combined PCL and ACL reconstruction (PCL-R/ACL-R), based on data from the Swedish National Knee Ligament Registry (SNKLR). It was hypothesized that patients undergoing isolated primary PCL-R and combined PCL-R/ACL-R are older and predominantly male, with less concurrent meniscus and cartilage injuries but a higher proportion of neurovascular injuries compared to patients undergoing primary isolated ACL-R.

## Materials and methods

Ethical approval was obtained from the Swedish Ethical Review Authority (registration number: 2020-03559). The study was performed in accordance with the Declaration of Helsinki.

The present study is a registry-based cohort study using data from the SNKLR. Since its foundation in 2005, the SNKLR aims to prospectively collect patient- and surgeon-reported data on operatively treated ACL and PCL injuries [1]. Demographic information, variables related to surgical techniques, ligamentous or articular injuries in conjunction with cruciate ligament tears, and additional operative findings are documented in the surgeon-reported section. Concomitant surgical procedures are documented as separate events linked to the primary injury. Patients have the right to request exclusion from the SNKLR in writing, thereby making participation in the SNKLR optional.

### Data collection and eligibility criteria

**Fig. 1:**
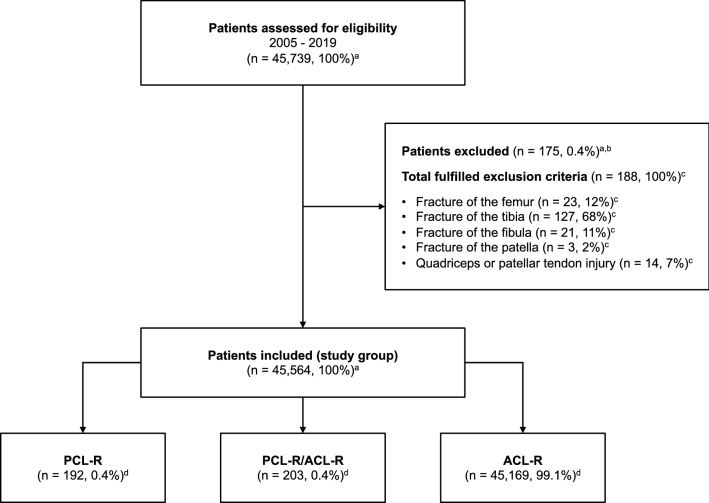
Flowchart of patient enrollment. Values are presented as count (*n*) and proportion (%). ^a^Percentage of patients assessed for eligibility; ^b^Some patients fulfilled more than one exclusion criteria; ^c^Percentage of total fulfilled exclusion criteria; ^d^Percentage of patients included in the study group; *ACL-R* anterior cruciate ligament reconstruction, *PCL-R* posterior cruciate ligament reconstruction, *PCL-R/ACL-R* combined posterior cruciate ligament reconstruction and anterior cruciate ligament reconstruction

Data collected between January 1, 2005 and December 31, 2019 were obtained from the SNKLR. Three study groups were created based on the injury and surgical procedure, that including patients undergoing primary isolated PCL-R, primary isolated ACL-R, and primary combined PCL-R/ACL-R. Exclusion criteria comprised fracture of the femur, fibula, patella or tibia and quadriceps or patellar tendon injury (Fig. 1). Demographic information such as age, sex, body mass index (BMI), injury laterality, time from injury to surgery, and mechanism of injury based on activity type was extracted for each patient. Injury mechanisms were categorized as sports-related, traffic-related, and other (other, outdoor activity, and work). Sports-related mechanisms were further subdivided into pivoting (American football/rugby, basketball, dancing, floorball, football, gymnastics, handball, ice hockey/bandy, martial arts, racket sports, volleyball, and wrestling), non-pivoting (cross-country skiing, cycling, horseback riding, motocross/enduro, skateboarding, snowboarding, and surfing/wakeboarding), and other physical activity (other recreational sport, exercise, and trampoline). Moreover, the extracted surgical data included the following: presence of (yes/no) concomitant injury to the medial and lateral menisci, articular cartilage injury regardless of International Cartilage Repair Society (ICRS) grade [4] based on intraarticular location, neurovascular injury, treatment of medial and lateral meniscus tear by repair or resection, treatment of articular cartilage injury by debridement, or microfracture and concurrent ligament injuries.

### Statistical analysis

Statistical analysis was performed using SPSS for Windows (Version 27.0. Armonk, NY: IBM Corp) and Stata Statistical Software (Release 16. College Station, TX: StataCorp LLC). Descriptive data are presented as the mean ± standard deviation (SD) and median with interquartile range (IQR). For categorical variables, count (*n*) and proportion (%) are reported. A Welch One-Way Analysis of Variance (ANOVA) analysis with Games–Howell post hoc pairwise comparison was conducted to compare patient groups with respect to continuous variables, such as age, BMI, and time from injury to surgery (in months). Assumptions for ANOVA were evaluated using Levene’s test for homogeneity of variances and a plot of residuals by fitted values. As the distribution of time between injury and surgery was heavily skewed (but not age and BMI), the non-parametric Kruskal–Wallis test was applied with Bonferroni correction for the post hoc comparisons. Values are therefore presented as median and IQR. Chi-squared test was used for comparisons between categorical variables, such as sex, right-sided injury laterality, injury mechanism, and all variables regarding concomitant injuries and treatments. When Chi-square test was significant, a post hoc pairwise comparison was performed with Bonferroni correction to adjust for the family-wise error using cellwise residuals [15]. These tests were carried out to compare individual cells within contingency. A two-tailed *p* value < 0.05 was considered significant.

## Results

### Patient demographics

**Fig. 2:**
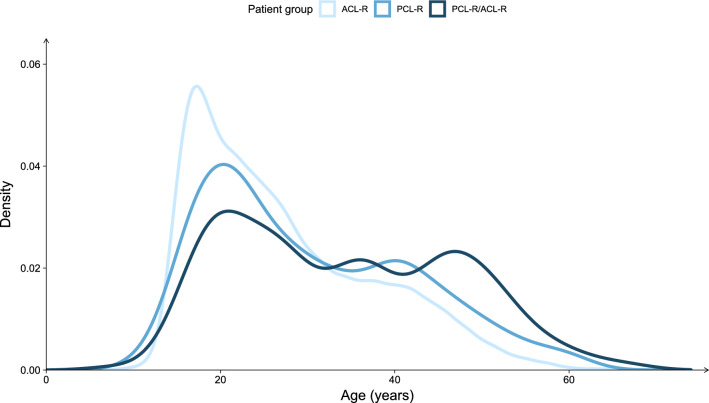
Density plot displaying the age distribution of patients undergoing isolated PCL-R, combined PCL-R/ACL-R, and isolated ACL-R at the time of surgery. Patients undergoing isolated PCL-R and combined PCL-R/ACL-R tend to be older compared to isolated ACL-R patients. *ACL-R* anterior cruciate ligament reconstruction, *PCL-R* posterior cruciate ligament reconstruction, *PCL-R/ACL-R* combined posterior cruciate ligament reconstruction and anterior cruciate ligament reconstruction

A total of 45,564 patients were registered with a primary cruciate ligament reconstruction in the SNKLR, of which primary isolated PCL-R was performed in 192 (0.4%), combined PCL-R/ACL-R in 203 (0.4%) and primary isolated ACL-R in 45,169 (99.1%) patients. The mean age at the time of ligament reconstruction significantly differed between groups and was 30 (SD ± 11.8), 34 (SD ± 12.9) and 27 (SD ± 10.4, *p* < 0.001) years for isolated PCL-R, combined PCL-R/ACL-R and isolated ACL-R patients, respectively (Fig. 2). The proportion of males was 8% greater in the combined PCL-R/ACL-R group compared to the isolated ACL-R group (*p* = 0.02), but no difference was observed among PCL-R and PCL-R/ACL-R groups. Median time from injury to surgery was significantly different between all groups (*p* < 0.001), with the combined PCL-R/ACL-R group having the shortest time (7 months, IQR 1–14 months), and isolated PCL-R having the longest average time until operative treatment (18 months, IQR 9–31 months). A detailed summary of the demographic characteristics of each patient group is reported in Table 1.

**Table 1:** Comparison of demographics and injury mechanism in patients treated with isolated PCL-R, combined PCL-R/ACL-R, and isolated ACL-R

Variable	PCL-R	PCL-R/ACL-R	ACL-R	*p* value
Number of patients, *n* (%)	192 (0.4)	203 (0.4)	45,169 (99.1)	
Age, [years]	30 ± 11.8^a,b^	34 ± 12.9^c^	27 ± 10.4	< 0.001*
Males, *n* (%)	115 (60)	131 (65)^d^	25,537 (57)	0.047**
BMI, [kg/m^2^]	25 ± 3.4	27 ± 5.5^e^	25 ± 3.4	< 0.001*
Right knee, *n* (%)	82 (43)	95 (47)	23,530 (52)	0.01
Time from injury to surgery, [months]	18 (9–31)	7 (1–14)	8 (4–18)	< 0.001***
Injury mechanism				< 0.001**
Sports-related, *n* (%)	122 (64)	109 (54)	40,085 (89)^f,g^	
Alpine/skiing, *n* (%)	12 (6)	22 (11)	6510 (14)	
Pivoting sport, *n* (%)	69 (36)	44 (22)	29,879 (66)	
Non-pivoting sport, *n* (%)	32 (17)	26 (13)	1934 (4)	
Other physical activity, *n* (%)	9 (5)	17 (8)	1762 (4)	
Traffic-related, *n* (%)	38 (20)^c^	54 (27)^c^	817 (2)	
Other, *n* (%)	32 (17)^e^	40 (20)^c^	4,182 (9)	

Values are presented as count (*n*) and proportion (%) for number of patients, male sex, right-sided laterality and injury mechanisms. Age and BMI are presented as mean ± SD. Time from injury to surgery is displayed as median and IQR. Between group differences were analysed using *Welch One-Way Analysis of Variance with Games-Howell correction for multiple pairwise comparisons, **Chi-square test with a post hoc column pairwise comparison using Bonferroni correction or ***Kruskal–Wallis test with a post hoc column pairwise comparison using Bonferroni correction. Injury mechanism in the ACL-R patient group is missing 85 values

*ACL-R* anterior cruciate ligament reconstruction, *BMI* body mass index, *IQR* interquartile range, *PCL-R* posterior cruciate ligament reconstruction, *PCL-R/ACL-R* combined posterior cruciate ligament reconstruction and anterior cruciate ligament reconstruction, *SD* standard deviation

^a^*p* < 0.01 vs. PCL-R/ACL-R, ^b^*p* < 0.01 vs. ACL-R, ^c^*p* < 0.001 vs. ACL-R, ^d^*p* < 0.05 vs. ACL-R, ^e^*p* < 0.01 vs. ACL-R, ^f^*p* < 0.001 vs. PCL-R, ^g^*p* < 0.001 vs. PCL-R/ACL-R

### Injury mechanism

**Fig. 3:**
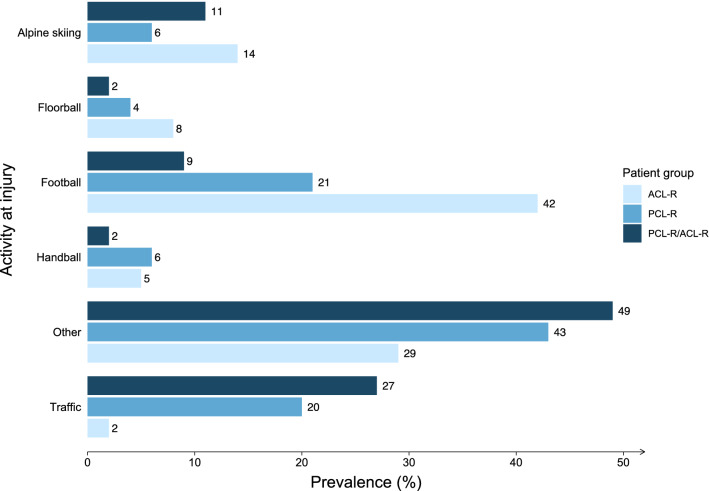
Alpine skiing, floorball, and football are frequent sports-related causes of injury in patients undergoing isolated PCL-R, combined PCL-R/ACL-R and isolated ACL-R. Less prevalent activities are categorized as “Others”. *ACL-R* anterior cruciate ligament reconstruction, *PCL-R* posterior cruciate ligament reconstruction, *PCL-R/ACL-R* combined posterior cruciate ligament reconstruction and anterior cruciate ligament reconstruction. Distribution of the most frequent activity types leading to injury in patients undergoing isolated PCL-R, combined PCL-R/ACL-R, and isolated ACL-R. Less prevalent activities are categorized as “Others”. *ACL-R* anterior cruciate ligament reconstruction, *PCL-R* posterior cruciate ligament reconstruction, *PCL-R/ACL-R* combined posterior cruciate ligament reconstruction and anterior cruciate ligament reconstruction

Sports-related activities were the most frequently reported mechanism of injury leading to isolated PCL-R (64%), combined PCL-R/ACL-R (54%), and isolated ACL-R (89%; Fig. 3). Football (21% vs. 9%) and alpine skiing (6% vs. 11%) were the two main contributing sports leading to subsequent isolated PCL-R or combined PCL-R/ACL-R. The reporting of a traffic-related injury mechanism was more prevalent in combined PCL-R/ACL-Rs and isolated PCL-Rs compared to isolated ACL-Rs (27% vs. 20% vs. 2%, *p* < 0.001). Of the injuries leading to isolated ACL-R in this study, 89% were sustained during sports. Figure 3 summarizes patient groups according to the activity type leading to injury.

### Concomitant injuries

The prevalence of any concomitant meniscal, chondral, or neurovascular injury was significantly lower in the isolated PCL-R group (44%) compared to the combined PCL-R/ACL-R (59%) and isolated ALC-R (56%) groups (*p* < 0.01; Table 2). Meniscus injury prevalence differed significantly among isolated PCL-R, combined PCL-R/ACL-R, and isolated ACL-R patient groups (16% vs. 31% and 45%, *p* < 0.001), as well as the distribution of lateral and medial meniscus injury (*p* < 0.001, Table 2). Significantly fewer patients with medial meniscus injury were treated by repair in conjunction to PCL-R as opposed to both combined PCL-R/ACL-R and isolated ACL-R (6% vs. 43% and 25%, *p* < 0.001, Supplemental Table 1). Resection was performed at a 25% greater rate for the treatment of medial meniscus injuries in the isolated PCL-R compared to the isolated ACL-R patient group (*p* = 0.01). Repair of the lateral meniscus was performed in 36% of patients with combined PCL-R/ACL-R compared to 17% in isolated ACL-Rs (*p* < 0.01).

**Table 2:** Concomitant injuries in patients treated with isolated PCL-R combined PCL-R/ACL-R and isolated ACL-R

Variable	PCL-R (*n* = 192)	PCL-R/ACL-R (*n* = 203)	ACL-R (*n* = 45,169)	*p* value
Concomitant injury, *n* (%)	85 (44)	120 (59)^a^	25,132 (56)^b^	< 0.01
Meniscus injury, *n* (%)	31 (16)	62 (31)^b^	20,190 (45)^c,d^	< 0.001
Lateral meniscus injury, *n* (%)	16 (8)	36 (18)^a^	11,425 (25)^c,e^	< 0.001
Medial meniscus injury, *n* (%)	17 (9)	37 (18)^a^	12,136 (27)^c,e^	< 0.001
Cartilage injury, *n* (%)	71 (37)	82 (40)	11,775 (26)	< 0.001
Lateral femoral condyle, *n* (%)	12 (6)	21 (10)^f^	2424 (5)	< 0.01
Medial femoral condyle, *n* (%)	52 (27)^f^	67 (33)^g^	8042 (18)	< 0.001
Lateral patella, *n* (%)	12 (6)^f^	17 (8)^g^	1218 (3)	< 0.001
Medial patella, *n* (%)	26 (14)^g^	27 (13)^g^	2147 (5)	< 0.001
Lateral tibial plateau, *n* (%)	17 (9)	22 (11)^h^	2807 (6)	< 0.01
Medial tibial plateau, *n* (%)	19 (10)^f^	33 (16)^g^	2274 (5)	< 0.001
Trochlea, *n* (%)	13 (7)^f^	24 (12)^g^	1315 (3)	< 0.001
Neurovascular injury, *n* (%)	2 (1)	18 (9)	12 (0.3)	< 0.001
Ligament injury, *n* (%)				
LCL, *n* (%)	12 (6)	71 (35)	363 (0.8)	< 0.001
MCL, *n* (%)	16 (8)	89 (44)	1616 (4)	< 0.001
PCL, *n* (%)	–	–	183 (0.4)	–
PLC, *n* (%)	5 (3)	56 (28)	53 (0.1)	< 0.001

Values are presented as count (*n*) and proportion (%) if not otherwise stated. Between group differences were analysed using the Chi-square test with a post hoc column pairwise comparison using Bonferroni correction

*ACL-R* anterior cruciate ligament reconstruction, *LCL* lateral collateral ligament, *MCL* medial collateral ligament, *PCL-R* posterior cruciate ligament reconstruction, *PCL-R/ACL-R* posterior cruciate ligament reconstruction and anterior cruciate ligament reconstruction, *PLC* posterolateral corner

^a^*p* < 0.05 vs. PCL-R, ^b^*p* < 0.01 vs. PCL-R, ^c^*p* < 0.001 vs. PCL-R, ^d^*p* < 0.001 vs. PCL-R/ACL-R, ^e^*p* < 0.05 vs. PCL-R/ACL-R, ^f^*p* < 0.01 vs. ACL-R, ^g^*p* < 0.001 vs. ACL-R, ^h^*p* < 0.05 vs. ACL-R

The prevalence of articular cartilage injury in combined PCL-R/ACL-R was 3% higher compared to isolated PCL-R/ACL-R (40% vs 37%). In contrast, isolated ACL-reconstructed knees displayed a significantly lower prevalence of concomitant cartilage injuries, present in 26% of cases (*p* < 0.001). The most common location of cartilage injury was the medial femoral condyle for all patient groups. The prevalence of cartilage injuries of the medial femoral condyle was significantly higher in the isolated PCL-R and combined PCL-R/ACL-R patient groups than that of the isolated ACL-Rs (27% vs. 33% and 18%, *p* < 0.001). Among isolated PCL-R and combined PCL-R/ACL-R groups, no difference was observed in the prevalence of medial or lateral patellar and medial tibial plateau cartilage injury. Cartilage injuries at the same locations were significantly less prevalent in the isolated ACL-R compared to the PCL-R and PCL-R/ACL-R patient groups (*p* < 0.001, Table 2). Operative treatment of cartilage injuries was performed at a rate of 0–26% across all patient groups, most often involving debridement on the medial femoral condyle (Supplemental Table 1).

Neurovascular injuries occurred at a significantly different rate among all groups (*p* < 0.001) and were present in 9% of combined PCL-R/ACL-R compared to 1% in isolated PCL-R and 0.3% in isolated ACL-R patients. Evaluation of concomitant ligament injuries among patient groups revealed medial collateral ligament (MCL), lateral collateral ligament (LCL), and posterolateral corner (PLC) injuries to be present in 44%, 35%, and 28% of knees treated by combined PCL-R/ACL-R, respectively. The presence of these concomitant ligamentous injuries was significantly different (*p* < 0.001 for each specific ligament) across all groups (Table 2).

## Discussion

**Fig. 4:**
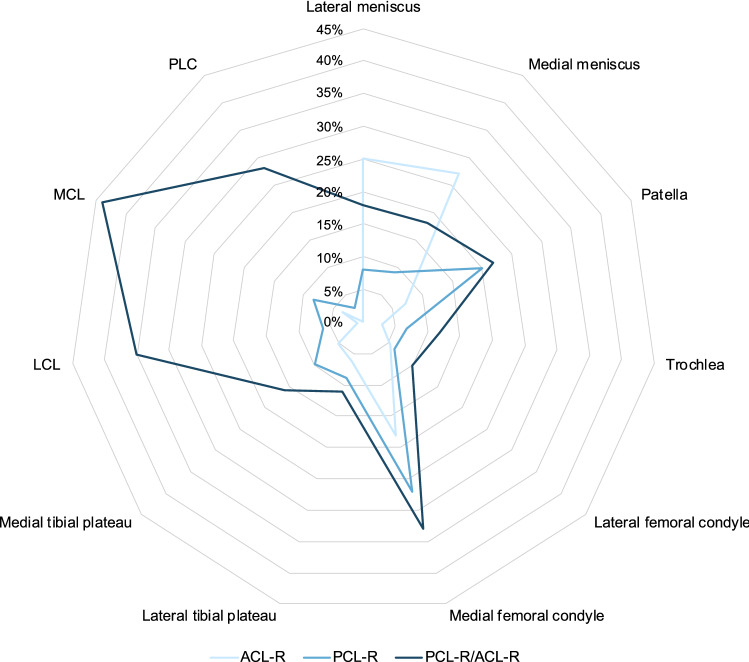
Patients undergoing isolated PCL-R, combined PCL-R/ACL-R and isolated ACL-R display different patterns of concomitant knee injuries. Patella, trochlea, medial femoral condyle, lateral femoral condyle, medial tibial plateau, and lateral tibial plateau refer to cartilage injuries at the respective locations. *ACL-R* anterior cruciate ligament reconstruction, *LCL* lateral collateral ligament, *MCL* medial collateral ligament, *PCL-R* posterior cruciate ligament reconstruction, *PCL-R/ACL-R* combined posterior cruciate ligament reconstruction and anterior cruciate ligament reconstruction, *PLC* posterolateral corner

The key finding of this study was the difference in the patterns of concomitant injuries among patients with isolated PCL-R, combined PCL-R/ACL-R, and isolated ACL-R (Fig. 4). Patients undergoing isolated PCL-R displayed a lower combined rate of concomitant meniscal, chondral, and neurovascular injuries (44%) compared to patients treated either by combined PCL-R/ACL-R (59%) or isolated ACL-R (56%). The results of the study highlight several differences between operatively treated isolated PCL and combined PCL/ACL tears in terms of demographics, injury mechanism, and associated knee injuries.

The demographic characteristics of the investigated patient groups are in agreement with the previous reports, as we further demonstrate that combined cruciate ligament reconstruction patients are generally older than both isolated PCL-R and ACL-R patients [14, 16, 21] and that there are a greater proportion of males undergoing PCL-R/ACL-R compared to isolated ACL-R (65% vs. 57%). The latter finding suggests the more frequent involvement of males compared to females in traffic accidents with high-energy trauma resulting in serious knee injury [14, 16]. The significantly longer median time from injury to surgery in the isolated PCL-R group compared to other groups is explained by the initial non-operative management of isolated PCL injuries often recommended in Scandinavia, followed by an optional delayed PCL-R if bracing and rehabilitation are unsuccessful [18, 23]. Longer preoperative times have been reported to result in more cartilage lesions in PCL-based isolated and combined knee ligament injuries, which may provide an explanation for the comparable proportions of cartilage injuries reported in patients requiring isolated and combined PCL-R in this study [11, 24].

Findings with respect to the injury mechanism of operatively treated PCL tears are concordant with previous Scandinavian knee ligament registry studies, highlighting their predominantly sports-related causes [14, 16]. This provides further evidence that earlier research [7, 22] conducted on small patient populations and in trauma settings may have overestimated the role of traffic-related accidents in the etiology of PCL tears. While sports are responsible for the majority of operatively treated cruciate ligament tears across all patient groups (54–89%), the role of traffic-related mechanisms are more pronounced in PCL-R and PCL-R/ACL-R patients (20% and 27%, respectively). The higher prevalence of traffic-related mechanisms in injuries with operative treatment of both cruciate ligaments compared to isolated tears further supports the notion that these tears require mechanisms with greater energy at trauma compared to those affecting only the PCL.

With respect to concomitant intraarticular injuries involving the articular cartilage and menisci, this study demonstrated a greater proportion of cartilage injuries in the patient groups with isolated (37%) or combined (40%) reconstruction of the PCL compared to the isolated ACL-R group (26%). High-energy associated direct forces to the knee during PCL tears may be a contributing factor to the higher prevalence of articular cartilage lesions observed in these patients. However, a recent investigation from the Danish Knee Reconstruction Registry reported a lower overall rate of cartilage lesions concomitant to PCL-R compared to ACL-R, discordant with the present findings [14]. Of the cartilage injuries in this study, medially localized injuries of the patella and femoral condyle were more frequent in knees requiring PCL-R in contrast to those where only the ACL was reconstructed, which is a pattern reported by earlier studies of PCL-injured knees [9, 19, 24]. Although increase in contact pressure exerted on the medial compartment of the isolated or combined PCL-deficient knee has been implicated in the development of cartilage injury over time [8, 24], the differences in mechanisms leading to PCL and ACL tears may also explain this characteristic contrast in cartilage injury patterns. Moreover, the overrepresentation of neurovascular (9%) and additional ligamentous (28–44%) injuries in the combined PCL-R/ACL-R group further highlights the detrimental role of high-energy forces and direct trauma to the knee, resulting in complex injuries. Conversely, the lower rate of meniscus injury reported in PCL-R patients may be due to the effect of posterior tibial translation during trauma, which unloads the posterior horns of the menisci and prevents injury at this location. Additionally, the rotatory component characteristic of ACL tears may play a less prominent role in the mechanism of PCL tears, resulting in a comparatively lesser strain on the menisci.

The treatment of concurrent meniscus and cartilage injuries in this study displays considerable variation among patient groups. While a higher rate of both medial and lateral meniscus resection compared to repair was reported across all groups, medial meniscus tears accompanying combined PCL-R/ACL-R were repaired at a higher rate compared to those in isolated PCL-R. The converse relationship was demonstrated regarding lateral meniscus injuries, although these differences did not reach statistical significance. Recent registry-based studies aimed at determining the effect of additional meniscus repair or resection concomitant to ACL-R on postoperative patient outcomes report contrasting results [5, 12, 25]. Consequently, the impact of meniscus injury treatment on functional outcomes following knee ligament surgery remains to be clarified. Despite the high prevalence of articular cartilage damage across all groups in this study, only a small proportion of cartilage injuries were treated operatively. The majority of these injuries remained either untreated or treatment was unreported. Postoperative patient-reported outcomes following PCL-R have previously been reported to be inferior compared to those of ACL-R, which may potentially be influenced by the presence and the treatment of concomitant meniscus and cartilage injuries [3, 14, 17].

While PCL tears are frequently the result of trauma involving high-energy forces such as dashboard injuries and falls on the flexed knee [22], ACL tears are almost exclusively caused by sports-related activity involving internal rotation of the tibia, pivoting, and valgus forces [2]. In the current study, injuries requiring reconstruction of both the PCL and ACL were frequently accompanied by additional tears of the MCL, LCL, or PLC. A recent investigation of combined PCL-Rs involving both the PCL and ACL similarly reported high rates of concurrent MCL (47%) and LCL/PLC (48%) tears [21]. However, the same study reported markedly greater rates of concomitant ligament injuries in isolated PCL-Rs compared to the present study [21]. While the previous research has drawn attention to the frequent incidence of PLC tears in conjunction with PCL tears [13, 21], the present study demonstrates a greater injury rate of the PLC and collateral ligaments in patients requiring combined PCL-R/ACL-R. Furthermore, these results are novel with regards to the high prevalence of concurrent MCL tears in the combined PCL-R/ACL-R patient group (44%) [16, 21]. The high degree of knee instability caused by bicruciate tears and the high-intensity forces in their injury mechanism may potentially explain the frequent injury of the MCL, LCL, and PLC in this subset of patients. While both isolated and combined PCL-R groups contain knees with tears of multiple knee ligaments, the two groups are firmly distinguished by their concomitant injury patterns. This information suggests that dividing combined injuries into subgroups based on the torn knee ligament pattern may be important to consider when assessing outcomes following operatively treated isolated and combined PCL tears.

The main strength of this study is that the SNKLR contains information on the activities leading to cruciate ligament tears and provides a detailed record of concomitant injuries. With a coverage of 90% of all ACL-Rs conducted in Sweden [1] and a large sample population, the registry enables the reliable comparison of the epidemiology of operatively managed PCL and ACL tears in Sweden. A limitation of this study is that the SNKLR is a surgical registry, which does not systematically collect information from patients with non-operatively treated cruciate ligament injuries. Consequently, the registry-based comparison of epidemiologic differences between operatively and non-operatively treated PCL injuries is currently not feasible. Thus, interpretation of study results is restricted to the context of operatively managed PCL tears. Additionally, the occasional incompleteness of registry data may lead to a misrepresentation of concomitant injuries and their treatment. The current study is based on a Swedish population, leading to some uncertainty with respect to the generalizability of the results. Finally, the reason for operative treatment of the PCL tears in this study is not provided in the registry, subjecting the observed results in this population to confounding by indication.

## Conclusions

Patients treated with isolated PCL-R, combined PCL-R/ACL-R, and isolated ACL-R display differences in concomitant meniscal and chondral injuries and their management. Differences in injury mechanisms may impact concomitant knee injury patterns observed among the patient groups. Cartilage and ligament injuries were more frequent in patients with injury to the PCL when compared to the ACL. Consequently, combined PCL and ACL tears should raise suspicion for concomitant intraarticular lesions with clinical relevance during the operative treatment of these complex injuries.

## Supplementary Information

Below is the link to the electronic supplementary material.

Supplementary file1 (DOCX 46 KB)

## Abbreviations

ACL: Anterior cruciate ligament
ACL-R: Anterior cruciate ligament reconstruction
ANOVA: Analysis of variance
BMI: Body mass index
IQR: Interquartile range
LCL: Lateral collateral ligament
MCL: Medial collateral ligament
PCL: Posterior cruciate ligament
PCL-R: Posterior cruciate ligament reconstruction
PLC: Posterolateral corner
SD: Standard deviation
SNKLR: Swedish National Knee Ligament Registry
