# Supplementary material for: Different injury patterns exist among patients undergoing operative treatment of isolated PCL, combined PCL/ACL, and isolated ACL injuries: a study from the Swedish National Knee Ligament Registry
Source: Knee Surg Sports Traumatol Arthrosc. 2022 Mar 31;30(10):3451–60. doi: 10.1007/s00167-022-06948-x (PMC9464165; doi:10.1007/s00167-022-06948-x)
Supplement: Supplementary file 1 — Supplementary file1 (DOCX 46 KB) [file 167_2022_6948_MOESM1_ESM.docx]

| Supplemental table 1. Surgical procedures in patients treated with isolated PCL-R combined PCL-R/ACL-R and isolated ACL-R | | | | |
| --- | --- | --- | --- | --- |
| **Variable** | **PCL-R**  **(n = 192)** | **PCL-R/ACL-R**  **(n = 203)** | **ACL-R**  **(n = 45,169)** | ***P* value** |
| **Lateral meniscus treatment**  (% of lateral meniscus injury)  Repair, n (%)  Resection, n (%) | 4 (25)  8 (50) | 13 (36)^a^  23 (64) | 1,905 (17)  7,656 (67) | <0.01  0.33 |
| **Medial meniscus treatment**  (% of medial meniscus injury)  Repair, n (%)  Resection, n (%) | 1 (6)  15 (88)^d^ | 16 (43)^b,c^  17 (46) | 3,034 (25)  7,533 (63) | <0.001  0.01 |
| **Cartilage treatment**  (% of Cartilage injury) |  |  |  |  |
| **Lateral femoral condyle**  Debridement, n (%)  Microfracture, n (%) | 1 (8)  0 | 0  0 | 226 (9)  132 (6) |  |
| **Medial femoral condyle**  Debridement, n (%)  Microfracture, n (%) | 10 (19)  3 (6) | 14 (21)  3 (5) | 1,256 (16)  602 (8) |  |
| **Lateral patella**  Debridement, n (%)  Microfracture, n (%) | 2 (17)  0 | 0  0 | 124 (10)  1 (0.1) |  |
| **Medial patella**  Debridement, n (%)  Microfracture, n (%) | 3 (12)  0 | 1 (4)  0 | 202 (10)  8 (0.4) |  |
| **Lateral tibial plateau**  Debridement, n (%)  Microfracture, n (%) | 0  0 | 0  0 | 74 (7)  14 (1) |  |
| **Medial tibial plateau**  Debridement, n (%)  Microfracture, n (%) | 2 (11)  0 | 0 (9)  0 | 131 (6)  21 (1) |  |
| **Trochlea**  Debridement, n (%)  Microfracture, n (%) | 1 (8)  0 | 0  0 | 112 (9)  70 (5) |  |
| Values are presented as count (n) and proportion (%) if not otherwise stated. Between group differences were analysed using Chi-square test with a post hoc column pairwise comparison using Bonferroni correction.  ACL-R = anterior cruciate ligament reconstruction; PCL-R = posterior cruciate ligament reconstruction; PCL-R/ACL-R = posterior cruciate ligament reconstruction and anterior cruciate ligament reconstruction  ^a^*p* < 0.01 vs. ACL-R,  ^b^*p* < 0.05 vs. PCL-R, ^c^*p* < 0.05 vs. ACL-R, ^d^*p* < 0.05 vs. PCL-R/ACL-R | | | | |
